# Supplementary figures and images for: Thermoregulatory heat-escape/cold-seeking behavior in mice and the influence of TRPV1 channels
Source: PLoS One. 2022 Nov 16;17(11):e0276748. doi: 10.1371/journal.pone.0276748 (PMC9668124; doi:10.1371/journal.pone.0276748)

# VMPO

Ta = 28°C

CON

CAP

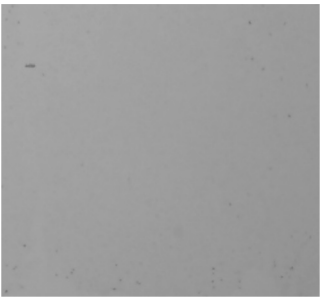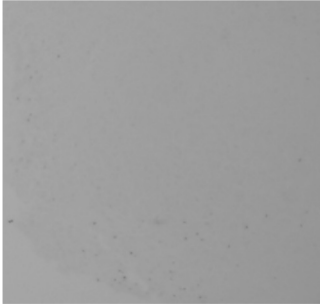

Ta = 37°C

CON

CAP

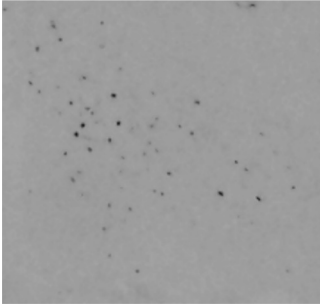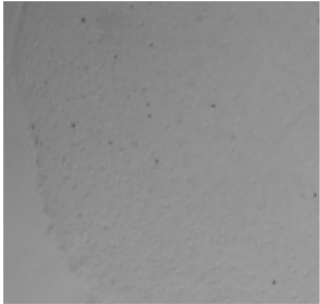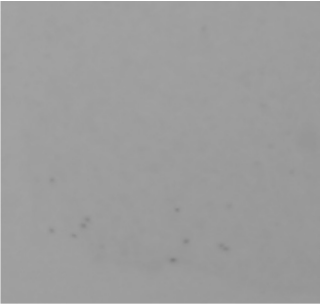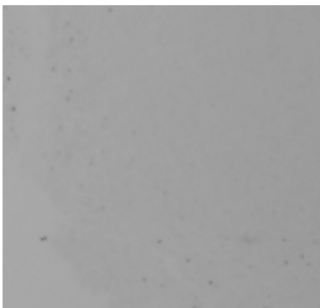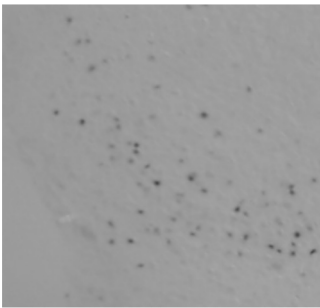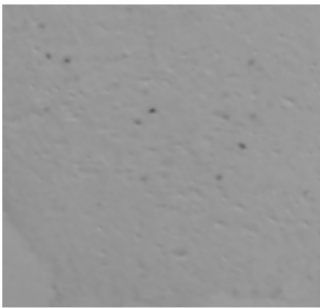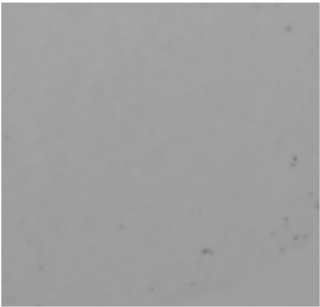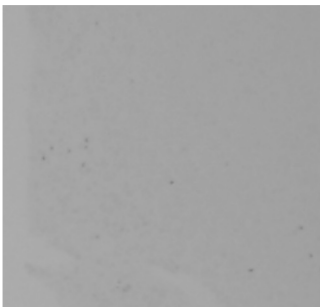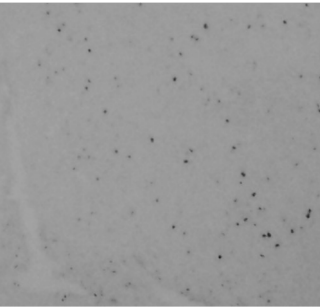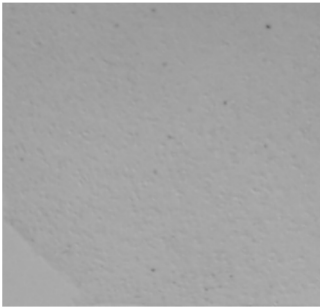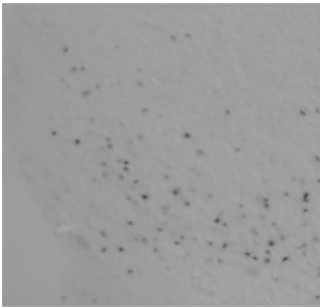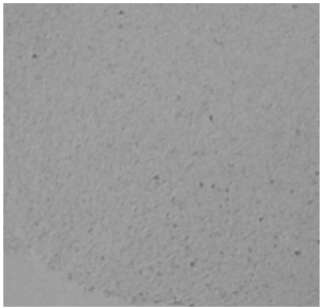

# MnPO

Ta = 28°C

CON

CAP

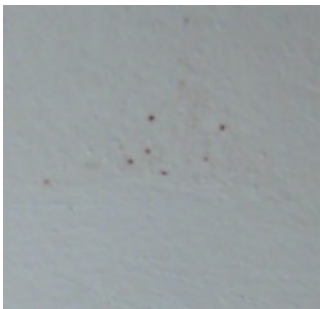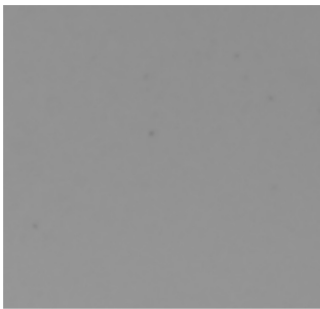

Ta = 37°C

CON

CAP

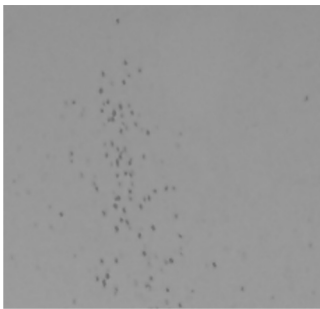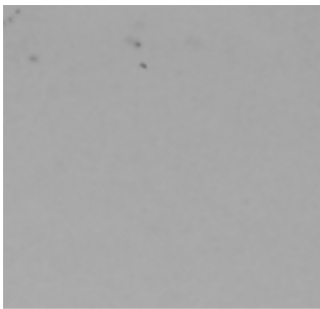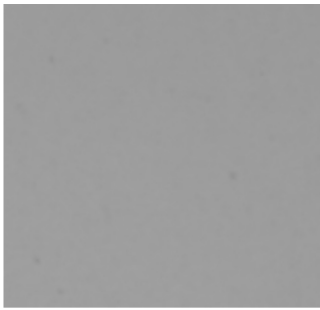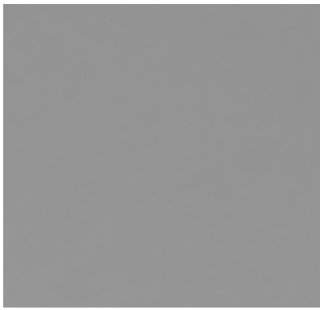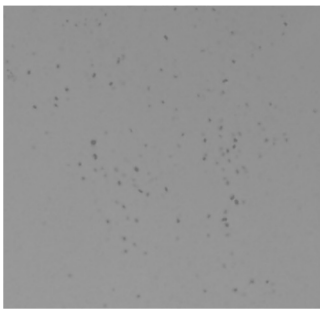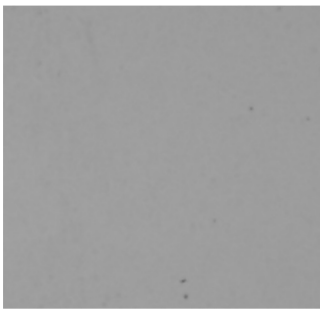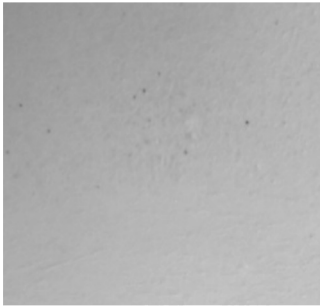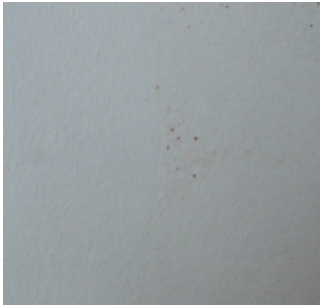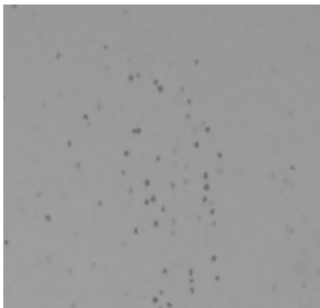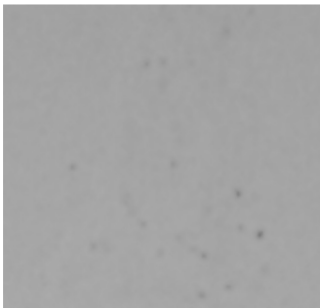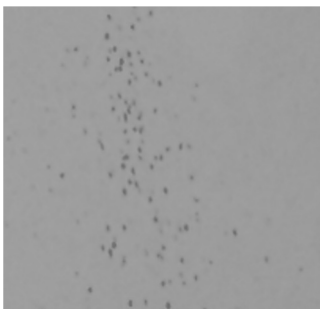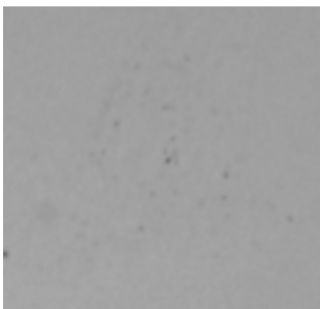

MPO

Ta = 28°C

CON

CAP

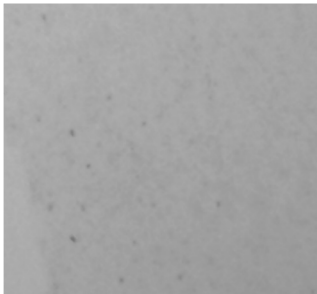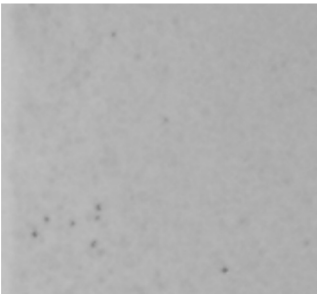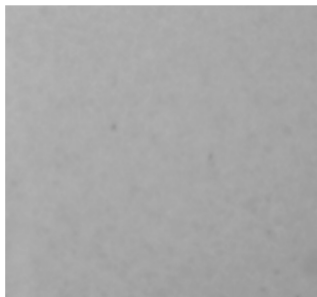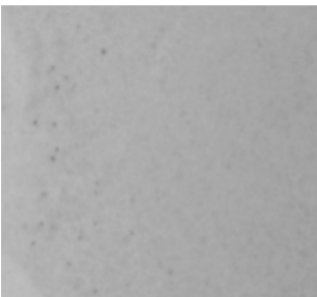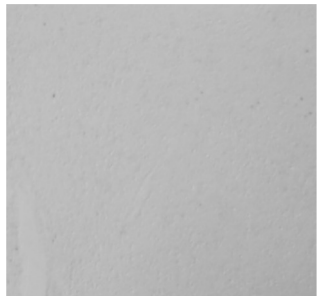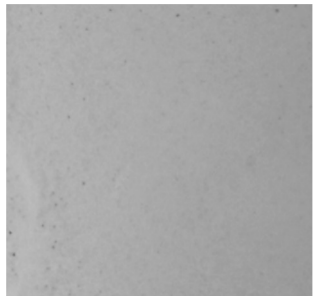

Ta = 37°C

CON

CAP

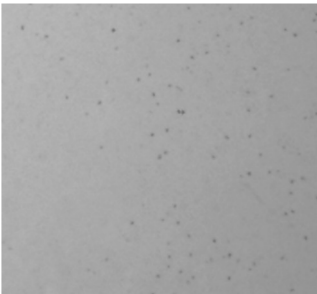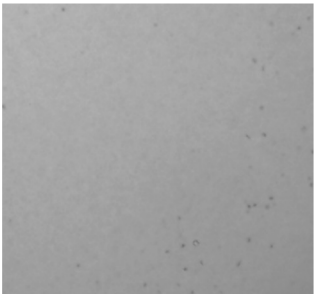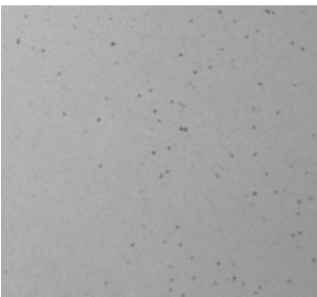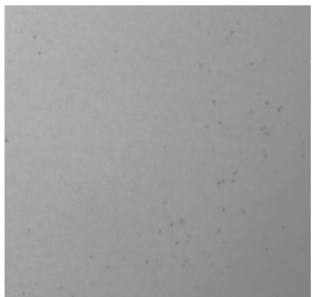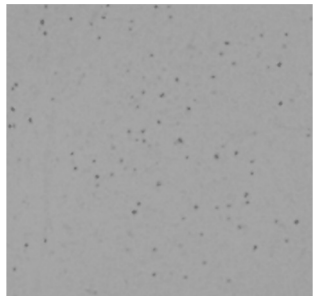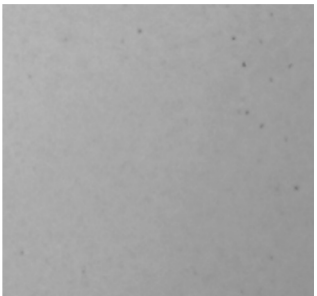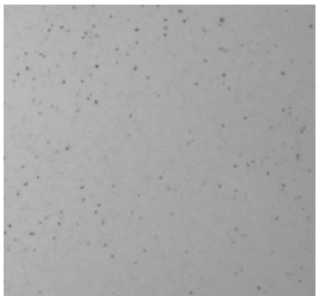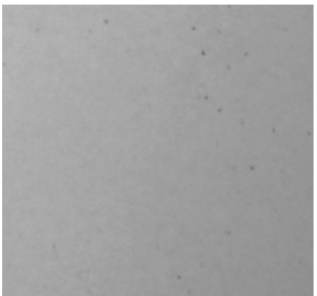

Supplement: S1 Fig — (PDF) [file pone.0276748.s001.pdf]
