# Supplementary material for: Thermoregulatory heat-escape/cold-seeking behavior in mice and the influence of TRPV1 channels
Source: PLoS One. 2022 Nov 16;17(11):e0276748. doi: 10.1371/journal.pone.0276748 (PMC9668124; doi:10.1371/journal.pone.0276748)
Supplement: Attachment — Submitted filename: Manuscript Number PONE-D-22-13073.docx [file pone.0276748.s003.docx]

**Title:**	Thermoregulatory Heat-Escape/Cold-Seeking Behavior of Mice and the Influence of Capsaicin Desensitization.

**Manuscript number:**	PONE-D-22-13073

The present study assessed heat-escape/cold-seeking behavior during thermoregulation and the role of TRPV1 channels in mice in this response. The desensitization was mediated by capsaicin administration and the behavioral thermoregulation was assessed using a newly developed system comprising temperature-controlled boards. The c-Fos expression in the preoptic area of the hypothalamus (POA) was evaluated after mice have been subjected to passive hyperthermia. The main results indicated that TRPV1 desensitized mice showed a more pronounced hyperthermia (in both experiments, i.e., during evaluation of behavioral thermoregulation when allowed to choose different Ta, and during passive hyperthermia when they were subjected to Ta = 37 °C); they also preferred environments with higher Ta values, indicating a suppression of behavioral thermoregulation (i.e., heat-escape/cold seeking behavior), resulting in hyperthermia. In addition, TRPV1 desensitized mice showed a lower neuronal activation in the POA in response to passive heating.

This study is relevant and brings new results about the role of TRPV1 channels in autonomic and behavioral thermoregulation. The methodology is quite interesting, however there are some points that should be better considered, mainly regarding to the results and interpretation and discussion of them.

Some considerations are below.

**Major points:**

1. In the “Introduction”, lines 70-71, it was described that “*It has been reported that systemic administration of larger doses of capsaicin results in the impairment of the hypothalamic function involved in thermoregulation*”. Does the dose of 50 mg/kg (2 injections) induce only peripheral or central and peripheral desensitization?
2. In the Experiment 1, the animals could choose between the most thermally comfortable quadrant, where the smallest value is Ta = 32 °C (data obtained in previous results). Wouldn't an increase in Tabd be expected even in this temperature condition in both groups (CON and CAP)? In the trial at 40 °C, it was observed that, in the first 30 min, the CON group remained ~ 70% in 32 °C and then (60-90 min) this percentage rose to 90%. Would this percentage difference (with the mice remaining ~ 20% longer in the 40 °C at the first minutes) contribute to at least a transient rise in Tabd?
3. In the Experiment 1, CAP group showed an increase in Tabd in all 3 trials (36, 38 and 40 °C). The animals showed this increase in Tabd because: a- they lost the drive of peripheral temperature recognition and did not perform behavioral thermoregulation more effectively by staying longer at higher Ta (passive hyperthermia) or b- (making an addendum with experiment 2) was there an impaired c-Fos expression in APO, which in turn, prevented autonomic thermoregulation to dissipate heat in the face of increased Tabd?
4. In the Experiment 2, to induce passive heating, it was used a Ta = 37 °C; however, the CON group showed no increase in Tabd. Did the test fail to induce hyperthermia? Why was a new temperature value not selected?
5. Overall, studies show that neuronal activation tends to be greater under conditions of increased core temperature. Interestingly, in this study, the CAP group, with higher values of Tabd, showed less neuronal recruitment and the authors attributed this response to the effect of capsaicin. Did the inhibition of neurons in APO by capsaicin prevent the activation of efferent pathways for heat dissipation, leading a more pronounced increase in Tabd?
6. The authors attributed a certain maintenance of autonomic thermoregulation for heat dissipation since there is a plateau for Tabd increase in the CAP group. Was any other brain area (that contribute to autonomic thermoregulation) with a lower density of TRPV1 channels that could be analyzed to verify if heat stress promoted a different effect (i.e., increased c-Fos expression) and thus, to conclude that capsaicin selectively induced an inactivation of thermoreceptors in the APO?

**Minor points:**

1. Capsaicin was used as a tool to desensitize the TRPV1 channels. Therefore, it may be more appropriate to replace the term “capsaicin” by “TRPV1” in the title and in other parts of the manuscript (i.e., Abstract, Aim…). For example: “Thermoregulatory Heat-Escape/Cold-Seeking Behavior of Mice and the Influence of TRPV1 channels”.
2. Abstract; Lines 21-22. “*Mice were administered a subcutaneous injection of capsaicin (50 mg/kg; CAP group) for desensitization or vehicle (control [CON] group)*”. Report what has been desensitized (TRPV1 channels).
3. Abstract; Lines 24-27. The description of the methodology in the abstract needs to be further specified. It seems that only 1 experiment was done. However, 2 different experiments were carried out; one to evaluate the behavioral thermoregulation (exposition to boards system) and the other one, for autonomic thermoregulation (exposition to Ta = 37˚C).
4. Abstract; Lines 27-31. To better understanding, results of experiment 1 (preference of different temperatures) and experiment 2 (changes in Tabd and c-Fos expression) should be describe separately.
5. Introduction; Lines 41-43. The authors show a study where animals with hypothalamic injury had impaired automatic response to cold but maintained the behavioral response; however, they concluded that “*this area of the brain may be responsible for these two different responses*”. The conclusion contradicts the result!
6. Introduction; Line 61. “*Studies have reported that local and/or systemic administration…*”. Specify which location; in the brain? Which area?
7. Introduction; Lines 65-67. The authors cited 2 controversial studies: “*It has been reported that capsaicin-desensitized rats prefer higher ambient temperature than normal rats (11)*” and “*Capsaicin-desensitized rats also showed avoidance behavior in 40°C heat* (no reference!!!)”. They did not provide an explanation for these contradictory results. Is it necessary to cite 2 controversial studies in this moment? Does this information contribute to elaborate the rationale behind the study question?
8. Introduction; Lines 74-77. I suggest merging sentences 1 and 2 in the same objective.
9. Introduction; Lines 76-77. “*We also compared the behavior of normal mice and capsaicin-desensitized mice*”. The sentence became repetitive.
10. Introduction; Lines 77-85. I suggest removing these sentences, once it is more applicated to “Methods” instead “Introduction”.
11. Methods; Lines 115-126. In the context of desensitization of TRPV1 channels, what is meaning of "eye wipes"? It is important to mention in the manuscript.
12. Methods; Line 154. Were the animals euthanized immediately after 30 minutes of passive heating? Was this time too short for evaluating the c-Fos expression?
13. Methods. It was not informed the coordinates used for sectioning the brain and the number of sections counted by area.
14. Results; Lines 184-186. The first two sentences can be removed once they correspond to the Methods. Suggestion: “The desensitization of TRPV1 channels was evaluated using the test of eye wipes stimulated by local application of capsaicin.”
15. Results; Lines 190-192; 197-200. These sentences refer to the Methods. I suggest removing it from "Results".
16. Figure 2. I suggest placing the legend at the top (next to graph A, instead of graph C). Inform N for each group.
17. Figure 4. I suggest inserting the Tabd records at the 28 °C. These data would help to confirm the influence of TRPV1 channels only under high temperature conditions.
18. Figure 5. The scale of the photoimages was not shown.
19. Figure 5; Graph C-b. The line that demonstrates the statistical difference is missing, as in the Graphs C-a and C-c.
20. Results; Lines 238-245. This information is best applied to the “figure legends”.
21. Figure 5. I suggest inserting the c-Fos expression at the 28 °C. Once again, these data would help to confirm the influence of TRPV1 channels only under high temperature conditions and it is important to evaluate the c-Fos expression in basal conditions and to compare with the stimuli (i.e., passive heating).
22. Inform the “N” in the legend of the figures and/or in the graphs.
23. Discussion; Lines 251-254. The first sentence alone does not seem to be an objective of the study. The main focus is the desensitization of TRPV1 channels. Therefore, I suggest merging the first 2 sentences to define the objective of the study.
24. Discussion; Lines 261-270. Re-evaluate the details showed in this section once they seem more applied to the “Methods” than to the “Discussion”.
25. Discussion, Lines 339-340. “*The number of cFos-IR cells in the POA of both groups increased after exposure to high ambient temperatures*”. This information was not cited in the “Results”. It was only reported that mice subjected to 28 °C showed less than 5; but it was not compared with the groups subjected to 37 °C.
26. Review typos. Some examples:
    - Line 33: “(*196 words*)”.
    - Line 38: “*are are*”.
    - Line 27 and Line 37: standardize POA 🡪 preoptic area of the hypothalamus (POA) / preoptic area (POA) of the hypothalamus.
    - Line 48: BDNF and PACAP were used only once in the manuscript. It is not necessary to use the acronym.
    - Line 53: “of >40°C” 🡪 > 40 °C (check space); “nociception..” (2 points).
    - Line 59: Yarmolinsky study was cited as reference of Caterina. Check references.
    - Line 83: “intensityof”.
    - Line 154: 1000 h.
    - Lines 220-221. “*Figure 3 shows Tabd during the control trial, the 36ºC trial (Fig 3A), the 38°C trial (Fig 3B), and the 40°C trial (Fig 3C) of the CAP and CAP groups*”. Description is not in accordance with the figure. The correct form should be as follows: control trial (Fig. 3A), *the 36ºC trial (Fig 3B), the 38°C trial (Fig 3C), and the 40°C trial (Fig 3D).*
    - Line 300: “hoewver".
    - Line 341: “han”.
