# Supplementary material for: Thermoregulatory heat-escape/cold-seeking behavior in mice and the influence of TRPV1 channels
Source: PLoS One. 2022 Nov 16;17(11):e0276748. doi: 10.1371/journal.pone.0276748 (PMC9668124; doi:10.1371/journal.pone.0276748)
Supplement: Attachment — Submitted filename: renamed_5cd31.docx [file pone.0276748.s004.docx]

August 26, 2022

Professor Samuel Penna Wanner

Academic Editor,

*PLOS ONE*

Dear Professor Samuel Penna Wanner,

Re: PONE-D-22-13073

Thank you very much for the thoughtful and constructive comments on our recently submitted manuscript titled “Thermoregulatory heat-escape/cold-seeking behavior in mice and the influence of TRPV1 channels”. We have revised the manuscript based on the comments and suggestions of the two reviewers. Our point-by-point responses to the reviewers’ comments are presented below.

We look forward to your feedback and to moving our manuscript closer to publication in *PLOS ONE*.

Kind regards,

Kei Nagashima, MBBS, DMSci

Body Temperature and Fluid Laboratory, Faculty of Human Sciences, Waseda University

Mikajima 2-579-15, Tokorozawa, Saitama 359-1192, Japan

Tel. & Fax: +81-4-2947-6918

E-mail: [k-nagashima@waseda.jp](mailto:k-nagashima@waseda.jp)

**Responses to Reviewer #1**

We thank the reviewer for the helpful comments and suggestions, which we have taken into consideration in our revised manuscript. The revised text is presented in blue in the manuscript.

*Major comments*

*1. In the “Introduction”, lines 70-71, it was described that “It has been reported that systemic administration of larger doses of capsaicin results in the impairment of the hypothalamic function involved in thermoregulation”. Does the dose of 50 mg/kg (2 injections) induce only peripheral or central and peripheral desensitization?*

>We conjecture that the desensitization was limited to the periphery, and the rationale for the conjecture in Ln 84-88, 410-413, and 415-416.

*2. In the Experiment 1, the animals could choose between the most thermally comfortable quadrant, where the smallest value is Ta = 32 °C (data obtained in previous results). Wouldn't an increase in Tabd be expected even in this temperature condition in both groups (CON and CAP)? In the trial at 40 °C, it was observed that, in the first 30 min, the CON group remained ~ 70% in 32 °C and then (60-90 min) this percentage rose to 90%. Would this percentage difference (with the mice remaining ~ 20% longer in the 40 °C at the first minutes) contribute to at least a transient rise in T_abd_?*

>We believe that the 32°C board did not provide a heat stimulus in both groups and added the rationale supporting this assumption in Ln 356 -358.

*3. In the Experiment 1, CAP group showed an increase in Tabd in all 3 trials (36, 38 and 40 °C). The animals showed this increase in Tabd because: a- they lost the drive of peripheral temperature recognition and did not perform behavioral thermoregulation more effectively by staying longer at higher Ta (passive hyperthermia) or b- (making an addendum with experiment 2) was there an impaired c-Fos expression in APO, which in turn, prevented autonomic thermoregulation to dissipate heat in the face of increased Tabd?*

>We conjecture that option (a) is correct. The reason is that i) the desensitization of TRPV1 channels was limited to the periphery (Ln 84-88, 410-413, and 415-416), and we evaluated the cFos expression in the brain regions which may reflect the thermal input from the periphery and/or the central temperature, not thermoregulatory responses (Ln 429-430 and 437-450). In this regard, we also address the limitations of the present study in Ln 475-479.

*4. In the Experiment 2, to induce passive heating, it was used a Ta = 37 °C; however, the CON group showed no increase in Tabd. Did the test fail to induce hyperthermia? Why was a new temperature value not selected?*

>We confirmed that Ta above 37ºC induced unrecoverable hyperthermia, which precluded the discussion on autonomic thermoregulatory function given the increase in T_abd_ in the CON group. We have added an explanation for using a heat stress of 37°C (Ln 423-425), and the lack of the temperature signal result in the increase in Tabd in the CAP group (Ln 84-88, 410-413, and 415-416).

*5. Overall, studies show that neuronal activation tends to be greater under conditions of increased core temperature. Interestingly, in this study, the CAP group, with higher values of Tabd, showed less neuronal recruitment and the authors attributed this response to the effect of capsaicin. Did the inhibition of neurons in APO by capsaicin prevent the activation of efferent pathways for heat dissipation, leading a more pronounced increase in Tabd?*

>We added the rationale for the decreased activation of cFos (Ln 428-429 and 436-450). We did not evaluate the cFos expression which may reflect the efferent thermoregulatory pathway. The limitation of the present study not to be able to answer to your inquiry was also added (Ln 474-478).

*6. The authors attributed a certain maintenance of autonomic thermoregulation for heat dissipation since there is a plateau for Tabd increase in the CAP group. Was any other brain area (that contribute to autonomic thermoregulation) with a lower density of TRPV1 channels that could be analyzed to verify if heat stress promoted a different effect (i.e., increased c-Fos expression) and thus, to conclude that capsaicin selectively induced an inactivation of thermoreceptors in the APO?*

>We focused on subregions in the POA that are associated with thermal input from the skin and did not consider other areas associated with an increase in central temperature. However, we cannot exclude the contribution of other brain areas, because the interaction of neural circuits involved in behavioral and autonomic thermoregulation remains unclear. We have added a relevant sentence accordingly (Ln 429-430, 437-451 and 475-479).

*Minor comments*

*1. Capsaicin was used as a tool to desensitize the TRPV1 channels. Therefore, it may be more appropriate to replace the term “capsaicin” by “TRPV1” in the title and in other parts of the manuscript (i.e., Abstract, Aim…). For example: “Thermoregulatory Heat-Escape/Cold-Seeking Behavior of Mice and the Influence of TRPV1 channels”.*

>We have replaced the term “capsaicin” by “TRPV1 channels” in the text as your suggestion suggested.

*2. Abstract; Lines 21-22. “Mice were administered a subcutaneous injection of capsaicin (50 mg/kg; CAP group) for desensitization or vehicle (control [CON] group)”. Report what has been desensitized (TRPV1 channels).*

>We have added this information to the revised text (Ln 22).

*3. Abstract; Lines 24-27. The description of the methodology in the abstract needs to be further specified. It seems that only 1 experiment was done. However, 2 different experiments were carried out; one to evaluate the behavioral thermoregulation (exposition to boards system) and the other one, for autonomic thermoregulation (exposition to Ta = 37˚C).*

>We have revised the sentences to clarify the methodology (Ln 23-34).

*4. Abstract; Lines 27-31. To better understanding, results of experiment 1 (preference of different temperatures) and experiment 2 (changes in Tabd and c-Fos expression) should be describe separately.*

>The results for the two experiments have been described separately (Ln 23-34).

*5. Introduction; Lines 41-43. The authors show a study where animals with hypothalamic injury had impaired automatic response to cold but maintained the behavioral response; however, they concluded that “this area of the brain may be responsible for these two different responses”. The conclusion contradicts the result!*

>We apologize for this error and have revised the sentence accordingly (Ln 49-52).

*6. Introduction; Line 61. “Studies have reported that local and/or systemic administration…”. Specify which location; in the brain? Which area?*

>The administration was conducted via the subcutaneous tissue, aiming to desensitize the peripheral neurons expressing TRPV1 channels. Thus, we rewrote the sentence in Ln 73-75.

*7. Introduction; Lines 65-67. The authors cited 2 controversial studies: “It has been reported that capsaicin-desensitized rats prefer higher ambient temperature than normal rats (11)” and “Capsaicin-desensitized rats also showed avoidance behavior in 40°C heat (no reference!!!)”. They did not provide an explanation for these contradictory results. Is it necessary to cite 2 controversial studies in this moment? Does this information contribute to elaborate the rationale behind the study question?*

>We wrote the rationale to cite the two studies and also added the missing reference in (Ln 77-82).

*8. Introduction; Lines 74-77. I suggest merging sentences 1 and 2 in the same objective.*

>The sentences have been merged as suggested (Ln 89-91).

*9. Introduction; Lines 76-77. “We also compared the behavior of normal mice and capsaicin-desensitized mice”. The sentence became repetitive.*

>The sentence has been removed.

*10. Introduction; Lines 77-85. I suggest removing these sentences, once it is more applicated to “Methods” instead “Introduction”.*

>We have removed these sentences as your suggestion.

*11. Methods; Lines 115-126. In the context of desensitization of TRPV1 channels, what is meaning of "eye wipes"? It is important to mention in the manuscript.*

We have revised this text to clarify the methodology and rationale for the behavior (134-146).

*12. Methods; Line 154. Were the animals euthanized immediately after 30 minutes of passive heating? Was this time too short for evaluating the c-Fos expression?*

>Animals were euthanized immediately after the 30-min heat. The period was determined based on the previous report (ref. 28). The part was changed (Ln 191-194).

*13. Methods. It was not informed the coordinates used for sectioning the brain and the number of sections counted by area.*

This information has been added to the text (Ln 207-213).

*14. Results; Lines 184-186. The first two sentences can be removed once they correspond to the Methods. Suggestion: “The desensitization of TRPV1 channels was evaluated using the test of eye wipes stimulated by local application of capsaicin.”*

>The two sentences have been removed and replaced as suggested.

*15. Results; Lines 190-192; 197-200. These sentences refer to the Methods. I suggest removing it from "Results".*

>We have removed the sentences as suggested.

*16. Figure 2. I suggest placing the legend at the top (next to graph A, instead of graph C). Inform N for each group.*

>We have placed the legend at the top and defined N in the figure legend.

*17. Figure 4. I suggest inserting the Tabd records at the 28 °C. These data would help to confirm the influence of TRPV1 channels only under high temperature conditions.*

>We added the data of T_abd_ during the exposure at T_a_ of 28°C in Fig 4.

*18. Figure 5. The scale of the photoimages was not shown.*

>The scale was inserted in the photoimages in Fig 5 in addition the information of the length was added in the legend (Ln 330).

*19. Figure 5; Graph C-b. The line that demonstrates the statistical difference is missing, as in the Graphs C-a and C-c.*

>The line has been inserted accordingly.

*20. Results; Lines 238-245. This information is best applied to the “figure legends”.*

>The information has been moved to the legend of Fig 5 as suggested.

*21. Figure 5. I suggest inserting the c-Fos expression at the 28 °C. Once again, these data would help to confirm the influence of TRPV1 channels only under high temperature conditions and it is important to evaluate the c-Fos expression in basal conditions and to compare with the stimuli (i.e., passive heating).*

>Due to limited space in the figures, we have added the photoimages in Fig 5 and newly added Fig 6, which illustrates the count analysis and provided graphs of the quantitative analysis (Ln 322-337).

*22. Inform the “N” in the legend of the figures and/or in the graphs.*

>"N” has been defined in the figure legends at all instances.

*23. Discussion; Lines 251-254. The first sentence alone does not seem to be an objective of the study. The main focus is the desensitization of TRPV1 channels. Therefore, I suggest merging the first 2 sentences to define the objective of the study.*

>The sentences have been merged and rewritten to clarify the main focus of this study (Ln 340-340).

*24. Discussion; Lines 261-270. Re-evaluate the details showed in this section once they seem more applied to the “Methods” than to the “Discussion”.*

>This text has been revised and moved to the methods section (Ln 154-158).

*25.	Discussion, Lines 339-340. “The number of cFos-IR cells in the POA of both groups increased after exposure to high ambient temperatures”. This information was not cited in the “Results”. It was only reported that mice subjected to 28 °C showed less than 5; but it was not compared with the groups subjected to 37 °C.*

>We have added relevant photoimages of cFos counts. Also, cFos quantitative analyses are presented in Fig 6 as a new graph.

*26.	Review typos. Some examples:*

*o	Line 33: “(196 words)”.*

*o	Line 38: “are are”.*

*o	Line 27 and Line 37: standardize POA  preoptic area of the hypothalamus (POA) / preoptic area (POA) of the hypothalamus.*

*o	Line 48: BDNF and PACAP were used only once in the manuscript. It is not necessary to use the acronym.*

*o	Line 53: “of >40°C”  > 40 °C (check space); “nociception..” (2 points).*

*o	Line 59: Yarmolinsky study was cited as reference of Caterina. Check references.*

*o	Line 83: “intensityof”.*

*o	Line 154: 1000 h.*

*o	Lines 220-221. “Figure 3 shows Tabd during the control trial, the 36ºC trial (Fig 3A), the 38°C trial (Fig 3B), and the 40°C trial (Fig 3C) of the CAP and CAP groups”. Description is not in accordance with the figure. The correct form should be as follows: control trial (Fig. 3A), the 36ºC trial (Fig 3B), the 38°C trial (Fig 3C), and the 40°C trial (Fig 3D).*

*>*Thank you for this helpful comment. The text has been corrected accordingly (Ln 275-276).

*o	Line 300: “hoewver".*

*o	Line 341: “han”.*

All the typos have been corrected.

**Responses to Reviewer #2**

We thank the reviewer for the helpful comments and suggestions, which we have taken into consideration in our revised manuscript. The revised text is presented in blue in the manuscript.

GENERAL COMMENTS

*1) The authors miss to mention relevant findings from research groups which contributed with meaningful achievements to the fields of behavioral thermoregulation and the involved neural structures; of the role of TRPV1 in thermoregulation in rodents; and of the use thermogradient systems. This ignorance resulted in incorrect statements and conclusions in many places throughout the manuscript.*

*Some major points are listed below:*

*i) the preoptic area is involved rather in autonomic than behavioral thermoregulation, while other brain areas such as the dorsal hypothalamic area. Was the c-Fos activity measured in that or other regions?*

>>We focused on subregions in the POA that are associated with thermal input from the skin and did not consider other areas associated with an increase in central temperature. However, we cannot exclude the contribution of other brain areas, because the interaction of neural circuits involved in behavioral and autonomic thermoregulation remains unclear. We have added a relevant sentence accordingly (Ln 429-430, 437-451 and 475-479). We also added a description for our assumption that the brain regions of our interest reflects thermal inputs from the skin surface (Ln 45-47).

*ii) while the exact role of TRPV1 in thermoregulation is still subject to research, some knowledge is already available about its function. For example, in rodents TRPV1 does not play a thermosensor role in thermoregulation, especially not in the periphery. Instead, TRPV1- expressing neurons in the MnPO mediate the effects of TRPV1 agonists and the same neuron population is presumably the one that is damaged by systemic vanilloid desensitization. Since the TRPV1-expressing MnPO neurons are within the warmth-defense thermoeffector pathway, their damage with systemic capsaicin desensitization can easily explain the bigger increase in core temperature in response to heat exposure, which was also observed by the authors in the present study. Because of the likely contribution of the damage of central TRPV1 channels to the observed changes, the discussion about the sole activation of cold-seeking behavior by peripheral heat signals (e.g., ln. 319-320; 336-338; and 354-356) should be rewritten.*

*Also, when discussing the role of TRPV1 in thermoregulatory responses in the hypothalamus and the effect of capsaicin desensitization on POA neurons, the authors should get more insight into the literature of the field (collected, for example, in PMID: 19749171). In sum, the discussion about the role of peripheral signals should be much more limited since it is not supported at all with any evidence (as also stated by the authors in ln. 369-371). On the other hand, the potential damage of the hypothalamic (MnPO) neurons could be emphasized more as it is supported with scientific literature.*

>We have revised our assertion in the text, given the limitation of explicitly stating that the phenomenon is purely due to peripheral desensitization, as you correctly pointed out. However, we believe that the peripheral effects of TRPV1 are controversial (Tan and Knight 2018). Indeed, after the study by Romanovsky et al. (2009), it has been demonstrated that peripheral TRPV1 agonist (capsaicin) administration activates warm-activated neurons in the POA (Tan et al. 2016). Furthermore, it has been reported that TRPV1 is very sparsely expressed centrally (in the brain) and to a lesser extent in the POA (Cavanaugh et al. 2011). Furthermore, the concentrations of capsaicin used in our study did not reach concentrations that would affect the central nervous system, as reported in previous studies (Pierau, Szolcsányi, and Sann 1986). We summarized these statements as a paragraph the discussion (Ln 430-464).

*In relation with the above, it would be important to check if the lower c-Fos expression was specific to heat exposure or could it be due to a loss of neurons induced by capsaicin. Can the desensitized mice increase their c-Fos activity similarly as controls in the POA in response to stimuli other than heat? In either case, since the mice could not use heat escape as behavioral thermoregulation in the c-Fos experiments (Experiment 2), it is hard to understand how the observed changes indicate alterations in heat escape/cold-seeking.*

>We did not evaluate if other stimuli could increase the cFos expression. Although we did not have direct evidence, it is unlikely that capsaicin caused the loss of central neurons, since the dose used in this study did not reach a dose that would have affected the central nervous system, as reported in previous studies (Ln 84-88). In addition, we did not find any influence on food intake, body weight gain, and spontaneous movement, which reflect hypothalamic function in a part at least (Ln 131-132).

*It is a further related point that it is already known that autonomic warmth-defense responses (e.g., skin vasodilation) to heat are impaired in capsaicin-desensitized animals, therefore statements about their potential contribution to the plateau of Tb should be rephrased (ln. 306- 309).*

>We have rephrased this statement and discussed how capsaicin desensitization affects autonomic thermoregulation (Ln 392-398 and 437-443).

*iii) when the authors compare their new system to previous devices (ln. 259-266), they mention that the corner preference was an obstacle in previous studies. However, they fail to mention a previously developed thermogradient system, which was repeatedly used for the study of behavioral thermoregulation (cold- and warmth-seeking) and could avoid the corner preference of the animals (see, for example, PMID: 16820025). The authors should also compare their new system to that one and familiarize with the results obtained in that system about brain nuclei of thermoregulatory behaviors.*

>We added the discussion in Ln 359-361.

SPECIFIC POINTS

*1) Ln. 46-49: the authors should not refer to a study published in 2016 as recent.*

>The term “recent” has been removed.

*2) Ln. 54-85: the last part of the sentence is unclear. Which two groups of mice experienced similar increases in body temperature?*

>The part showing the hypotheses in the present study was revised (Ln 89-93).

*3) The dose of capsaicin used for desensitization (50 + 50 mg/kg s.c.) is quite high. How was it tolerated by the mice? Did the authors use any intervention to help the recovery of the mice*

>The intervention to minimize circulatory distress which is often observed after the injection was shown in Ln 122 - 125. In addition, the tolerance for the injection was added in Ln 130 – 132.

*4) Why was it necessary to use different vehicle to dissolve capsaicin for desensitization and for the eye-wiping test?*

>The reason for the difference was because we followed the procedure for each previous report. We cited the respective study.

*5) Was always the same eye of the mice used in the eye-wiping experiments? Ln. 124: since the liquid were administered in a counterbalanced order, the application could not be randomized on the day of the second experiment.*

>We have rewritten the sentence to clarify the procedure in Ln 144-145.

*6) Ln. 130: What does “Each system” mean? Were more than one systems built and used in the experiments?*

>“Each” was replaced with “The” (Ln 151).

*7) Why was 32C set only in areas 2, 3, or 4, but not in area 1 during trials 1 to 3? Ln. 143-144: the authors state the order of the 4 trials was randomized, but later in Results this is contradicted as they state that the control trial was always the first. This should be explained.*

>We have revised the sentences to clarify that the trials, including the control trial, were randomly ordered (Ln 159-171). The reason for setting area 1 at 36°, 38°C, or 40°C was to assess the geographic effects. However, we did not observe the effects. This has been added to the discussion, and the underlying rationale has been incorporated in the methods (Ln 168-167 and 358-361).

*8) It is probably enough to state that the results are expressed in the mean and SD format in line 181. So, it could be deleted each time the results are specified to improve the readability of the text (e.g., ln. 187; 194-195, etc.).*

>The descriptions of SD have been removed.

*9) Ln. 215-217: was the resting time in area 5 also reduced compared to areas 1-4 in the desensitized mice?*

>We added a sentence indicating that the resting percentage at area 5 was also 0% and was less than that at other areas (Ln 270-272).

*10) Ln. 221-222: the sentence in its current form does not make sense, it should be corrected.*

>We corrected the sentence accordingly (Ln 275-276).

*11) Ln. 234: the text says the difference started at 5 min, but in Fig. 4 it is shown from 10 min. It should be corrected.*

>The part was corrected (Ln 299).

*12) Ln. 280-281: if we assume equal distribution among four areas, then why would be the ratio less than 25%? This does not make any sense. Accordingly, I find it misleading to discuss the results as if the increased warmth preference of the desensitized mice could be excluded (e.g., ln. 285-286), because that was not shown here. It was shown only that they spent less time than controls at 32C compared to 36, 38, and 40C. Similarly, I think it is also a false statement that their ability to discern thermal stimuli was completely abolished (ln. 294).*

>We revised the part to clarify the interpretation of the data (Ln 365-378).

*13) Ln. 344-346: “Another study” is mentioned, but both sentences refer to the same paper.*

>This was inadvertent error. We have replaced reference number 7 with number 8 (Tan CL, Cooke EK, Leib DE, Lin YC, Daly GE, Zimmerman CA, et al. Warm-sensitive neurons that control body temperature. Cell. 2016;167: 47–59.e15. doi: 10.1016/j.cell.2016.08.028.)

*14) Ln. 349-351: The sentence looks incorrect. Why would unchanged temperature in the control group mean that the number of c-Fos cells indicate response to thermal stimuli from the body surface?*

>We conjecture that option (a) is correct. The reason is that i) the densitization of TRPV1 channels was limited to the periphery (Ln 84-88, 410-413, and 415-416), and we evaluated the cFos expression in the brain regions which may reflect the thermal input from the periphery and/or the central temperature, not thermoregulatory responses (Ln 429-430 and 437-451). In this regard, we also address the limitations of the present study in Ln 475-479.

*15) Ln. 364-365: The sentence in its current form is false. Thermoregulatory behavior can be activated by many other stimuli than “heat information”, for example, capsaicin, endotoxin, etc.*

>The sentence has been reworded accordingly (Ln 470-472).

*16) The quality of representative photos in Figure 5A should be checked. In printed version hardly any structure can be recognized. Bar scale should be also inserted.*

>The images were replaced, and scale bars were inserted.

*17) The language of the manuscript should be substantially improved, e.g., ln. 38 “are are”; ln. 53 “..”; ln. 63 “TPPV1”; ln. 83: “intensityof”; ln. 221 “CAP and CAP groups”; ln. 242: “MnPO, MnPO”; ln. 253: capsicin; ln. 341 “han”, etc.*

>The text has been corrected accordingly, thank you.
