# Supplementary material for: Thermoregulatory heat-escape/cold-seeking behavior in mice and the influence of TRPV1 channels
Source: PLoS One. 2022 Nov 16;17(11):e0276748. doi: 10.1371/journal.pone.0276748 (PMC9668124; doi:10.1371/journal.pone.0276748)
Supplement: Attachment — Submitted filename: REVIEW Manuscript Number PONE-D-22-13073_R1.docx [file pone.0276748.s005.docx]

**Title:**	Thermoregulatory Heat-Escape/Cold-Seeking Behavior of Mice and the Influence of Capsaicin Desensitization.

**Manuscript number:**	PONE-D-22-13073_R1

The present study assessed heat-escape/cold-seeking behavior during thermoregulation and the role of TRPV1 channels in mice in this response. The desensitization was mediated by capsaicin administration and the behavioral thermoregulation was assessed using a newly developed system comprising temperature-controlled boards. The c-Fos expression in the preoptic area of the hypothalamus (POA) was evaluated after mice have been subjected to passive hyperthermia. The main results indicated that TRPV1 desensitized mice showed a more pronounced hyperthermia (in both experiments, i.e., during evaluation of behavioral thermoregulation when allowed to choose different Ta, and during passive hyperthermia when they were subjected to Ta = 37 °C); they also preferred environments with higher Ta values, indicating a suppression of behavioral thermoregulation (i.e., heat-escape/cold seeking behavior), resulting in hyperthermia. In addition, TRPV1 desensitized mice showed a lower neuronal activation in the POA in response to passive heating.

Some considerations were made and it was gave to the authors the opportunity for reassessment. In the new update it was possible to verify a significant improvement in writing and in the discussion of the results. All “minor points” were corrected as requested, and the “major points” were better discussed.

The most important questions that still lack a plausible explanation are the fact that (1) the CON group did not show an increase in Tab at relatively high Ta, i.e., 32 °C (in the Experiment 1) and, mainly, 37 °C (in the Experiment 2); and (2) the reduction of neuronal activation in the APO in the CAP group even with the hyperthermic response.

To the first point, the authors informed that the Ta = 37°C was chosen to avoid unrecoverable hyperthermia caused by higher ambient temperature than 37°C and to evaluate thermoregulatory responses including autonomic system responses. I understand that this temperature value did not induce an increase in Tab in the CON group (unlike what was explained) and, even so, an autonomic response is expected in these conditions. So I think the “Experiment 2” failed to induce passive hyperthermia as proposed.

To the second point, the authors attribute the lower neuronal activation to the possible reduction of afferent inputs that were reduced by peripheral desensitization of TRPV1 channels, considering that the capsaicin treatment would not change the central TRPV1 channels. It would be important to evaluate other hyperthermia-responsive nuclei to verify a possible increase in activation and confirm the hypothesis.

However, throughout the text, the authors included such limitations in the study.

**Minor points:**

1. Introduction (line 47): After citing reference 4, start the text with a new paragraph and introduce a link to start contextualizing behavioral thermoregulation. "On the other hand, in the behavioral thermoregulation, the heat-escape cold-seeking behavior is a key…"
2. Introduction (lines 51 and 54): I suggest that the conclusions of cited studies follow in the same sentence of the description of the results.
3. Introduction (lines 77 and 79): It seemed confusing to inform the values of Ta in parentheses as if they were referring to the rats.
4. Legend of Figure 5: The symbol † does not appear in the graph. Remove it of legend.
5. Legend of Figure 5: Change “Ab, d and f” 🡪 “Ab, f and j”.
